# Demographic synchrony increases volatility and extinction risk in human populations

**DOI:** 10.1017/ext.2026.10013

**Published:** 2026-05-05

**Authors:** Marcus John Hamilton, Robert Walker

**Affiliations:** 1 Anthropology, The University of Texas at San Antonio, USA; 2 Santa Fe Institute, USA; 3 Center for Data Science, University of Texas at San Antonio, San Antonio, USA; 4 Anthropology, University of Missouri, USA; 5 Center for Applied Statistics and Data Analysis, University of Missouri, Columbia, USA

**Keywords:** vulnerability, stability and collapse, prehistoric, population size, demographic stochasticity

## Abstract

Cooperation is widely viewed as a stabilizing force because it pools risk and buffers against environmental uncertainty. Demographic theory predicts that volatility in per-capita population growth declines rapidly with population size, implying that larger populations should be more stable. Yet human societies of all sizes eventually collapse. Here, we propose a general mechanism that helps explain this pattern. We show that cooperation generates demographic synchrony—correlated survival and reproductive outcomes among individuals—which inflates aggregate volatility. Using more than 200 longitudinal population records from indigenous societies in the Brazilian Amazon, we find that volatility declines with population size substantially slower than predicted under demographic independence. This pattern implies pervasive within-population correlation, such that census populations behave demographically as if composed of far fewer independent modules. We formalize this process with a synchrony-extended stochastic model in which shared environmental exposure, social coupling, and spatial overlap generate demographic correlations that erode the stabilizing effects of scale. We term this dynamic the *cooperation–synchrony paradox*: the same institutions that reduce risk for individuals can increase vulnerability to population-wide shocks. Further, we propose this is likely a general mechanism across species, where cooperation stabilizes locally but generates systemic fragility by reducing effective demographic dimensionality.

## Impact statement

Cooperation is one of the most powerful strategies in the evolution of life. By sharing resources, coordinating behavior and pooling risk, cooperative organisms, from social insects to human societies, reduce individual vulnerability to environmental uncertainty. Classical demographic theory, therefore, predicts that larger populations should become increasingly stable because random fluctuations in births and deaths average out as populations grow. Yet the historical and ecological record repeatedly contradicts this expectation. Large populations sometimes experience dramatic demographic collapses despite appearing robust. This study addresses that paradox. Drawing on more than 200 time series of indigenous populations in the Brazilian Amazon, we show that population volatility declines with size far more slowly than predicted by classical demographic models. This deviation indicates that individuals within populations are not demographically independent. Shared ecological conditions, cooperative institutions, social networks and spatial overlap synchronize survival and reproduction across individuals, producing correlated modular demographic outcomes within populations. We refer to this dynamic as the cooperation–synchrony paradox. Cooperation stabilizes individuals by reducing idiosyncratic risks, but it simultaneously couples demographic outcomes across group members. As these correlations increase, the effective number of independent demographic units in a population declines. Populations, therefore, fluctuate with fewer degrees of freedom, weakening the stabilizing effects of scale and increasing vulnerability to large shocks. These results have implications well beyond Amazonian populations. By linking human demography with ecological synchrony theory, the study reveals a general constraint on the resilience of cooperative systems. Population size alone does not guarantee stability when demographic fates are shared. Understanding how cooperation reshapes demographic dynamics is therefore essential for explaining the persistence, fragility and collapse of complex social systems across the tree of life.

## Introduction

Cooperation is one of the most powerful evolutionary strategies in the history of life (Nowak, 2006). Across taxa, cooperative organisms, from eusocial insects to cooperative breeding birds and mammals – including human societies – achieve ecological success through collective action (Krause and Ruxton, 2002; Rubenstein and Lovette, 2007; Schindler et al., 2010; Bowles and Gintis, 2011; Rubenstein, 2011; Clutton-Brock, 2016). By pooling resources, sharing risk and coordinating behavior, cooperation reduces individual variance in survival and reproduction and enables populations to persist in complex stochastic environments. Yet cooperative systems are also prone to sudden collapse: colonies fail, packs disappear and societies crash despite appearing demographically robust (Lande, 1993; Tainter, 1988; Morris and Doak, 2002; Diamond, 2011; Turchin, 2018). This raises a fundamental question that spans ecology, evolution and anthropology: how can cooperation both stabilize and destabilize populations?

The human past provides a dramatic illustration of this tension, where societies repeatedly grow in scale only to undergo demographic collapse from some combination of internal and external processes (Tainter, 1988; Currie et al., 2010; Diamond, 2011; Walker and Hamilton, 2011; Butzer, 2012; Hamilton et al., 2014; Walker and Hamilton, 2014; Hamilton and Walker, 2018). Indeed, societal collapse is a recurrent process in cultural evolution and plays a major role in human demographic history (Cumming and Peterson, 2017; Hamilton and Walker, 2018; Gurven and Davison, 2019). While collapse is often framed in political or institutional terms, its ultimate manifestation is a demographic sudden reduction in population viability. In this article, we use the term “collapse” strictly in this demographic sense to refer to situations where fluctuations in births, deaths or migration push populations below some critical threshold from which recovery is unlikely (Shaffer, 1981; Lande, 1993; Morris and Doak, 2002; Lande et al., 2003).

Classical demographic theory predicts that larger populations should be increasingly stable. If births, deaths and migrations are independent stochastic events, volatility in per-capita growth rates declines as $\sigma \left(r\right)\propto N^{-1/2}$ (Shaffer, 1981; Lande et al., 2003; Keyfitz and Caswell, 2005). Thus, under this assumption, the law of large numbers guarantees that scale generates stability by rapidly averaging out stochasticity, and so volatility declines with scale. Yet these population dynamics seem to be constantly violated as large populations collapse while small populations sometimes persist.

Ecologists have long recognized that spatial synchrony undermines stability (Lande et al., 1999). When populations fluctuate together due to the shared environmental forcing of the Moran effect, dispersal coupling, trophic interactions or shared pathogens (Koenig, 2002; Liebhold et al., 2004; Moran, 1953; Bjørnstad et al., 1999), regional extinction risk increases because rescue effects weaken (Brown and Kodric-Brown, 1977; Earn et al., 1998; Hanski, 1999). However, synchrony is typically studied among subpopulations within metapopulations, and far less attention has been given to synchrony *within* populations, particularly in cooperative organisms.

In cooperative systems, individuals do not experience demographic events independently. Individuals share resources, exposure to pathogens, reproductive opportunities and social networks. Cooperative breeding, shared childcare, collective foraging and coordinated mobility couple individual fates. These forms of social integration introduce correlations in survival and reproduction, increasing covariance among demographic outcomes. As covariance rises, populations no longer behave demographically as collections of independent individuals. Instead, populations fluctuate with fewer degrees of freedom.

We term this demographic tension the *cooperation–synchrony paradox.* Cooperation evolves to buffer individuals against risk, reducing idiosyncratic variance in fitness-related outcomes. Yet the mathematical consequence of pooling risk is the creation of shared exposures and shared outcomes, which build demographic synchrony. Synchrony inflates group-level volatility by reducing the effective demographic degrees of freedom in a population. In a synchrony-extended model, populations behave not as N independent individuals but as $K\left(N\right)$ effective demographic “clusters,” where K grows more slowly than N . As synchrony increases, the law of large numbers weakens, and volatility declines more slowly with scale than classical theory predicts. Cooperation stabilizes individuals while potentially increasing vulnerability at the population level by increasing modularity.

We investigate this paradox using a unique dataset of more than 200 time series of indigenous Amazonian populations compiled from Brazilian government censuses and historical records (see §3 for details). These small-scale societies represent modular, kin-structured and spatially bounded cooperative systems. Across 1,579 census observations and 1,353 growth intervals, we are able to measure volatility in per-capita growth rates and examine how stability scales with population size. These data provide a rare–perhaps anthropologically unique–opportunity to quantify demographic synchrony in small-scale human societies and to test whether the stabilizing effect of scale operates as classical models predict.

By bridging human demography with ecological theory, we argue that demographic synchrony represents a general constraint on the resilience of cooperative organisms. Just as synchrony among subpopulations elevates regional extinction risk in metapopulations, demographic synchrony among individuals elevates risk within single populations by inflating volatility. The Amazonian case serves not merely as a historical example of vulnerability, but as a tractable empirical system for understanding how cooperation reshapes the statistical structure of demographic processes.

First, we develop the mathematical framework for demographic synchrony. We then describe the data and estimation procedures and go on to present empirical estimates of volatility scaling and synchrony parameters and discuss data limitations. Finally, we explore the implications for cooperative organisms more broadly, arguing that demographic synchrony may represent an underappreciated source of fragility in social systems across the tree of life, and summarize these findings.

## The model

### Definitions and setup

We begin by defining variables where i indexes an individual population (or human society) and t indexes time steps (e.g., years). Let $N_{i,t}$ be a census size of the i th population at the time t . $A_{i,t}$ is the total geographic area associated with that population. It follows then that the population density of the i th population at the time t is $D_{i,t}\equiv N_{i,t}/A_{i,t}$ . The instantaneous growth rate of the i th population at the time t is then $r_{i,t}\equiv \ln\left(N_{i,t+1}/N_{i,t}\right)$ .

### Baseline demographic stochasticity

Under the demographic-noise baseline, individuals are assumed to act independently, and the annual probability of an individual reproducing is statistically independent. As such, net demographic increments are approximately Poisson with variance proportional to N (Shaffer, 1981). Let $\Delta N_{i,t}$ denote the net increment with variance $\mathrm{Var}\left(\Delta N_{i,t}\right)=v\;N_{i,t}$ . Since $r_{i,t}\approx \Delta N_{i,t}/N_{i,t}$ for small increments, we obtain the well-known scale-dependent relationship

(1) $$\mathrm{Var}\left(r|N\right)\approx \frac{v}{N}\propto N^{-1},\Rightarrow \sigma \left(r|N\right)\approx \sqrt{\frac{v}{N}}\propto N^{-1/2}.$$

Equation 1 is the demographic baseline expectation, where demographic volatility decays as $N^{-1/2}$ under demographic independence.

### A synchrony-extended model

The observed per-capita growth rate of a population is the average of many individual contributions. Let $y_{\mathrm{ij},t}$ denote the demographic contribution of the individual j in population i at time t to the change in population size between t and t+1 . Each $y_{\mathrm{ij},t}$ is a random variable representing the stochastic outcome of demographic processes such as survival, birth, death or migration. For instance, survival without associated change contributes $y_{\mathrm{ij},t}\approx 0$ , death or out-migration contributes $y_{\mathrm{ij},t}<0$ and birth or in-migration associated with the individual j contributes $y_{\mathrm{ij},t}>0$ .

Formally, we assume

$$E\left[y_{\mathrm{ij},t}\right]=\mu_{i},\;\mathrm{Var}\left(y_{\mathrm{ij},t}\right)=s^{2},$$

We introduce pairwise correlation $\rho_{i,t}$ across individuals within the same population:

$$\text{Corr}\left(y_{j,t},y_{k,t}\right)=\rho \;\text{for}\;j\neq k.$$

The per-capita growth rate is then the average of the individual demographic contributions across the population

(2) $$r_{i,t}=\frac{1}{N_{i,t}}\sum_{j=1}^{N_{i,t}}y_{\mathrm{ij},t}.$$

and the conditional variance of the per-capita growth rate in this synchrony-extended model is then

(3) $$\mathrm{Var}\left(r|N\right)=\frac{1}{N_{i,t}^{2}}\;\mathrm{Var}\left(\sum_{j=1}^{N_{i,t}}y_{\mathrm{ij},t}\right)=\frac{s^{2}}{N_{i,t}}\;\left[1+\left(N_{i,t}-1\right)\rho_{i,t}\right].$$

If $\rho_{i,t}=0$ , we recover the demographic baseline $\sigma \left(r\right)∼N^{-1/2}$ , whereas if $\rho_{i,t}>0$ , volatility falls more slowly with N .

### Statistical estimation of scaling parameters

We then wish to use data to estimate the parameters of the volatility scaling law we just derived:

(4) $$\sigma \left(r|N\right)=c\;N^{-\alpha }.$$

We proceed as follows. First, population sizes are grouped into exponentially increasing bins along the N -axis, with bin sizes $N_{\mathrm{bin}}=1,2,4,8,16,32,\ldots$ . Estimating the scaling relation given by equation 4 requires systematically measuring the change in standard deviation of the growth rate as the population increases in scale. Exponential binning of N ensures that each scale of population size is equally represented in the analysis. Because the distribution of N is typically right-skewed, with many small populations and relatively few large ones, equal-width bins would overweight small populations and underweight large ones. Exponential bins balance the representation of different scales, stabilize variance estimates within bins and ensure that the regression on lnN is not dominated by the smallest populations. This procedure is standard in scaling analyses of fluctuations (Stanley et al., 1996).

For each bin, we collect all observed growth rates $r_{i,t}$ from populations whose size $N_{i,t}$ falls within that bin, and compute the empirical standard deviation

$$\hat{\sigma }\left(r|N_{\mathrm{bin}}\right)=\sqrt{\mathrm{Var}\left\{r_{i,t}:N_{i,t}\in N_{\mathrm{bin}}\right\}}.$$

This yields a binned set of estimates across the population-size distribution. We then fit the log-linearized scaling relation

(5) $$\ln\hat{\sigma }\left(r|N\right)=\ln c-\alpha \ln N_{\mathrm{bin}}+\varepsilon ,$$

where lnc is the intercept, α is the slope and ε is an error term. Ordinary least squares regression of $\ln\hat{\sigma }\left(r|N\right)$ on lnN provides point estimates of c and α , while the dispersion of residuals provides confidence intervals. In this framework, c represents the baseline volatility at N=1 , and α quantifies the rate at which volatility decays with population size.

## Materials and methods

### Demographic data collection

We compiled our dataset from the (Instituto Socioambiental, 2025), including sources (Ricardo and Ricardo, 1986, 1991, 1996, 2000, 2006, 2011, 2016, 2023). These are population census estimates of recently contacted indigenous populations throughout Brazil collected over many generations. These sources provided 1,580 individual census estimates for 227 populations, with the earliest estimate from 1749 and the most recent from 2023. Population sizes range from single digits to tens of thousands, all of which would have experienced population crash events sometime in their history (Hamilton et al., 2014). Longitudinal data of indigenous population sizes gathered from direct census counts are incredibly rare in anthropology. These populations have primarily subsistence-based economies consisting of some combination of foraging, horticulture and agriculture. However, many of these populations will have experienced increasing interactions with market-based economies over time.

### Administrative census of contacted populations

Demographic data for indigenous populations in Brazil derive primarily from administrative census systems operated by federal agencies, particularly the Fundação Nacional dos Povos Indígenas (FUNAI) and the Special Secretariat of Indigenous Health (SESAI). These data are subsequently compiled and systematized by the Instituto Socioambiental (ISA) through its *Povos Indígenas no Brasil* (PIB) database. The resulting dataset represents a longitudinal administrative census system for contacted indigenous populations (and an indirect monitoring system for isolated [uncontacted] groups, not reported here, but see Walker and Hamilton, 2014, 2019 and Walker et al., 2023).

For contacted indigenous societies, demographic data are collected through direct village-level population counts. The basic unit of enumeration is the village, typically located within legally recognized indigenous Lands (*Terras Indígenas*). FUNAI regional offices and SESAI health districts (*Distritos Sanitários Especiais Indígenas*, DSEIs) maintain local population records that are updated during routine administrative visits, health campaigns and special interventions.

Village-level enumeration generally consists of complete counts of all residents physically present at the time of visit. Field agents record total population size and, in many cases, age and sex composition. These counts are transmitted to regional and national administrative databases. Because SESAI administers vaccination programs, primary care and epidemiological surveillance, health registries often function as continuously updated population lists.

There is no single synchronized national indigenous census cycle. Instead, the system operates as a rolling administrative census, with updates occurring at irregular intervals depending on logistical access, health activities and local conditions. National census operations conducted by the Brazilian Institute of Geography and Statistics (IBGE) provide additional cross-validation at decadal intervals, although discrepancies may arise due to seasonal mobility, urban migration and differences in ethnic self-identification.

Area estimates for populations also come from the Brazilian government and estimate the area assumed to be used by populations within indigenous protected areas, known as *Terras Indígenas.* These areas are not well-defined or demarcated, and may well overlap in many cases. Such areas will also be exploited in different ways, depending on the mobility and economics of each society. We do note, however, that despite measurement error, the relationship we report between area and population size for these Amazonian societies in these data is consistent with other more controlled studies of small-scale societies, where, in general, $AN^{\beta }$ and β<1 are often between 0.6 and 0.85 (Hamilton et al., 2007, 2020; Walker et al., 2014). Remarkably, we find similar spatial scaling relationships in a wide variety of human aggregations, including cities, both ancient and modern (Bettencourt et al., 2007; Ortman et al., 2020).

### Enumeration following sustained peaceful contact

For groups undergoing their first sustained peaceful contact, population counts are typically conducted during or immediately after contact missions. Emergency health teams conduct rapid enumeration to assess population size and vulnerability to infectious disease. Follow-up counts are often conducted at relatively short intervals during the first 5–20 years after contact, when mortality risks are elevated and demographic volatility is greatest. These early post-contact series are particularly important for estimating contact-related mortality and subsequent rebound dynamics (Hamilton et al., 2014).

### Data compilation and standardization

The ISA/PIB database functions as a centralized demographic repository. ISA compiles population figures from FUNAI, SESAI, IBGE and other official sources; standardizes ethnonyms; harmonizes geographic identifiers; and documents the year and source of each estimate. ISA does not conduct primary censuses but aggregates and systematizes official figures to produce publicly accessible time series. These data are released periodically and irregularly, as available.

### Data characteristics and limitations

The principal strengths of this system are (i) reliance on full village counts rather than statistical sampling for contacted populations, (ii) multi-decade longitudinal depth and (iii) integration with health and administrative infrastructures. However, limitations include variable update frequency across regions, potential undercounting in highly mobile populations, political variability affecting data transparency and the inherently indirect nature of estimates for isolated groups. Cross-border populations may also be incompletely enumerated when censuses do not include communities located outside Brazil.

Due to the extreme sensitivity of the data, specific locations of the villages and populations are not available, limiting the ability to control for potential spatial autocorrelation or to examine other important ecological processes such as mobility, interaction networks or spatial synchrony.

Despite these limitations, the administrative census framework provides a robust empirical foundation for reconstructing contact mortality, estimating minimum viable population sizes, modeling post-contact growth trajectories and evaluating long-term demographic resilience in indigenous Amazonia.

## Results

From the available data, we were able to calculate 1,353 instances of population growth. For each period of growth, we estimated the average annual growth rate over these time windows as

(6) $${\hat{r}}_{i,t}=\left(\frac{1}{\Delta t}\right)\ln\left(\frac{N_{i,t+\Delta t}}{N_{i,t}}\right).$$

**Figure 1.**
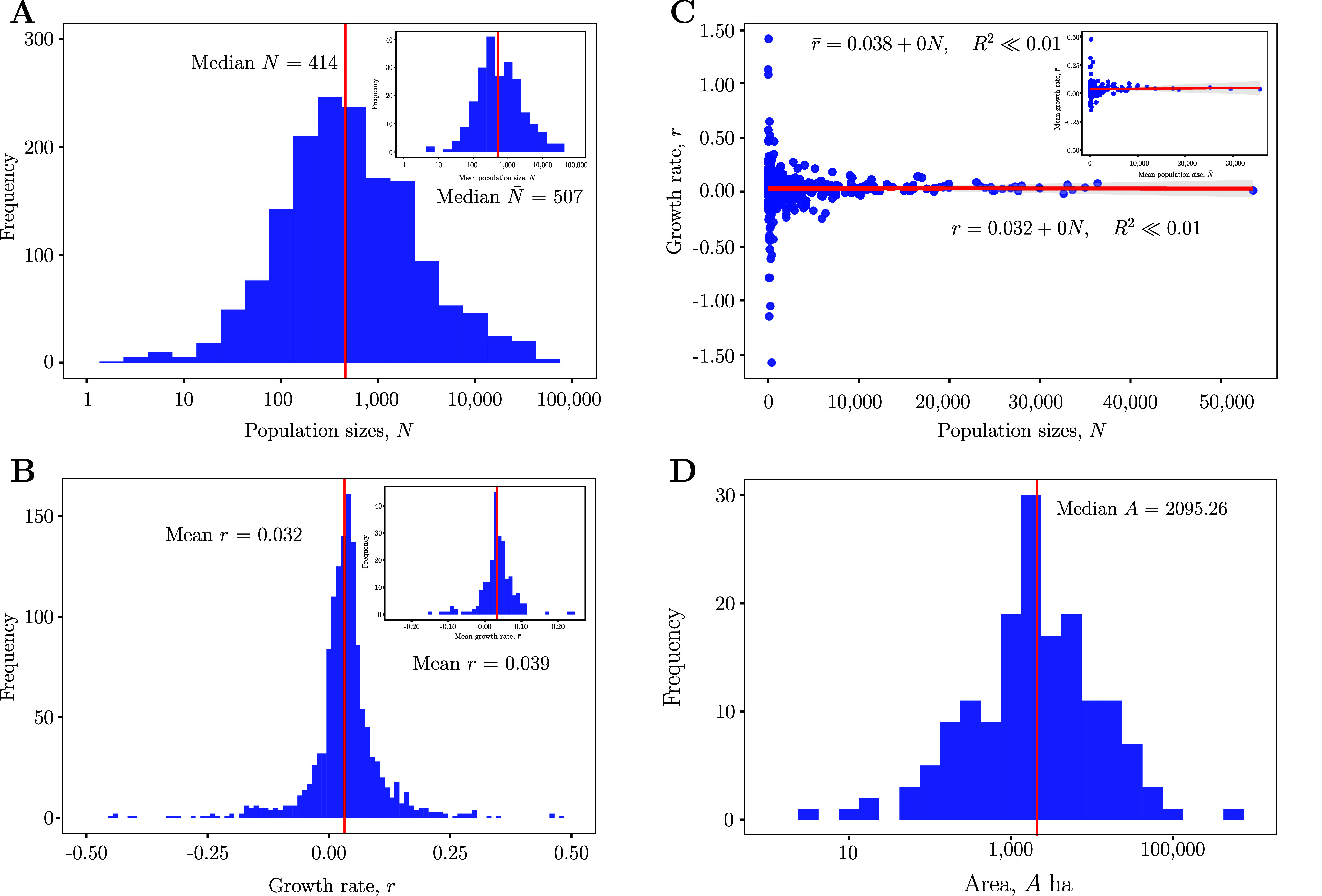
Population size and growth rates. (A) The distribution of all population size estimates from census populations is approximately lognormal with a median of 414 individuals. Inset in the upper right is the distribution of the average size of the 227 populations over their individual observation windows, with a median size of 507 individuals. (B) The distribution of all annual growth rates estimated from the census data (equation 6) with the mean growth rate $\bar{r}=0.032$ or 3% per year (see Table 1. for details). Inset is the average growth rate for each of the 227 populations over their individual observation windows, with a mean growth rate ${\bar{r}}_{N}=0.039$ or 4% per year. (C) Growth rate r as a function of population size N over all census estimates. The relationship shows a funnel plot typical of stochastic population dynamics where population volatility decreases with scale. Inset is the average growth rate of a population $r_{N}$ as a function of the average population size $\bar{N}$ . In both cases, population growth rates are not size-dependent. (D) The distribution of the geographic ranges, or areas A in hectares, for 161 of the 227 populations. The distribution is approximately lognormal with a median size of 2095.26 ha. Figure 1. long description.

Figure 1A shows the distribution of population size estimates across the data set, and the inset is the average population size for each of the 227 populations. These distributions are approximately lognormal, as might be expected for populations generated by a multiplicative growth process (Hamilton and Walker, 2018). The median population size over all estimates is 414, and the median is 507.

Figure 1B shows the distribution of growth rate estimates (equation 6) across the data set, and the inset is the average growth rate for each of the 227 populations. The distribution is leptokurtic and non-normal, though approximately symmetrical. The average growth rate over all estimates is $\bar{r}=0.032$ or 3.2% annual growth, and the median growth rate is $r_{N}=0.039$ , or 3.9% .

Figure 1C shows a funnel plot of the growth rate ${\hat{r}}_{i,t}$ as a function of the corresponding population size $N_{i,t}$ at the beginning of the growth period. The inset figure is a plot of the average per population. Fitted OLS regressions (red lines) summarized in Table 1 show that growth rates are independent of population sizes, but are much more volatile at small population sizes.

**Table 1.** Growth rate and population size scaling Table 1. long description.

	Dependent variable:
	*r*
${\hat{r}}_{i,t}$	–0.0000(−0.0000 , 0.0000)
Constant	0.03^*^(0.03 , 0.04)
Observations	1,353
*R* ^2^	0.0000
Residual std. error	0.12 (df = 1,351)
*F* statistic	0.004 (df = 1; 1,351)

*Note:* *p < 0.01

Figure 1D shows the distribution of territory size estimates A in hectares (equation 6) across the data set. The distribution is approximately lognormal. The median territory size across populations is 2095 ha.

**Figure 2.**
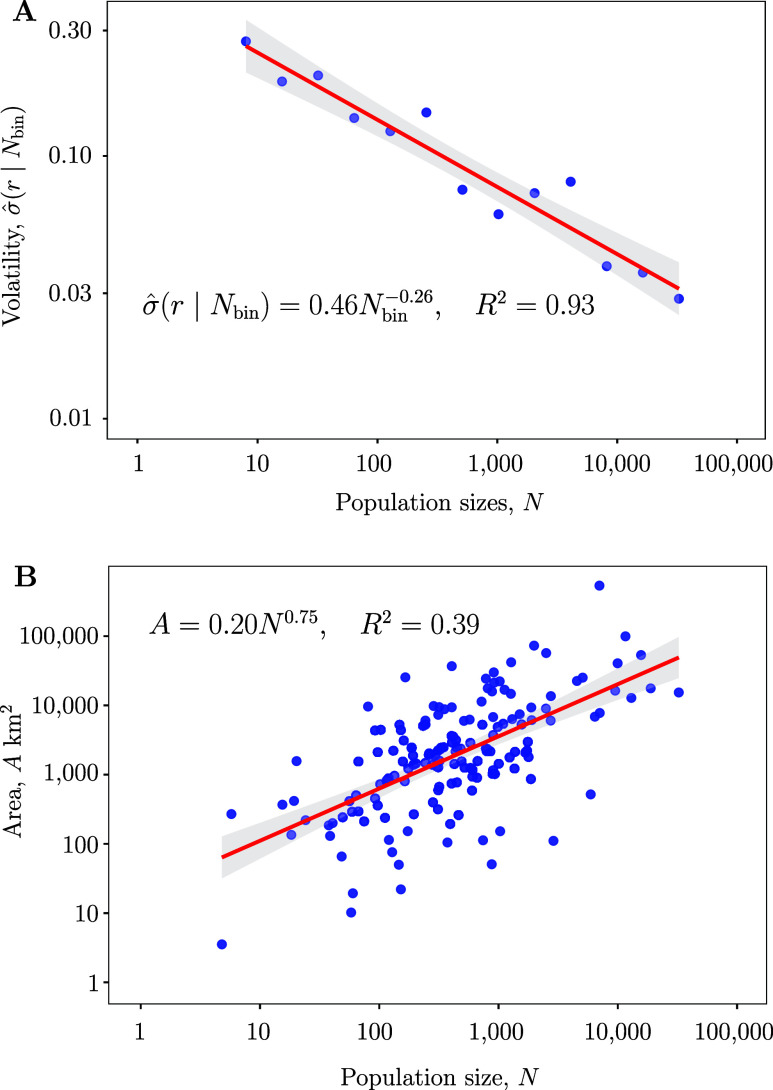
Demographic and population size area scaling. (A) Observed demographic volatility as a function of binned population size on a log-linear plot, following the methods outlined in the Methods section. Here, volatility declines with population size with a slope α=−0.26 (see Table 2. for details). (B) The scaling of the area use A by population size N for 161 of our 227 populations (limited by data availability) on log–log axes. An OLS estimate of the relationship yields a sublinear slope of β=0.75 (see Table 3. for details), as is commonly observed in the spatial ecology of traditional societies (Hamilton et al., 2007, 2012). Figure 2. long description.

Figure 2A shows the log–log plot of the conditional standard deviation of growth rates, the volatilities, $\hat{\sigma }\left(r|N_{\mathrm{bin}}\right)$ as a function of binned population sizes $N_{\mathrm{bin}}$ . A fitted OLS regression (red line) summarized in Table 2 shows that across populations, demographic volatility decreases with population size at a rate $\alpha =-0.26\left(-0.30,-0.21\right)$ .

**Table 2.** Sigma scaling Table 2. long description.

	Dependent variable:
	$\hat{\sigma }\left(r|N_{\mathrm{bin}}\right)$
α	–0.26^*^(−0.30 , –0.21)
lnc	–0.81^*^(−1.09 , –0.53)
Observations	13
*R* ^2^	0.93
Residual std. error	0.20 (df = 11)
*F* statistic	142.78 ${}^{*}$ (df = 1; 11)

*Note:* *p < 0.01.

Figure 2B shows the log–log plot of the area of the territory size of a population as a function of the population size. A fitted OLS regression (red line) summarized in Table 3 shows that across populations, area scales positively with population size at a rate β=0.75 .

**Table 3.** Area-population size scaling Table 3. long description.

	Dependent variable:
	lnA
β	0.75^*^(0.61 , 0.90)
Constant	2.99^*^(2.07 , 3.90)
Observations	160
*R* ^2^	0.39
Residual std. error	1.45 (df = 158)
*F* statistic	100.94 ${}^{*}$ (df = 1; 158)

*Note:* ${}^{*}$ *p* < 0.01.

### Synchrony scaling with N

Table 2 shows α=−0.26±0.05≈−1/4 and so empirically $\sigma \left(r\right)\propto N^{-1/4}\Rightarrow \mathrm{Var}\left(r\right)\propto N^{-1/2}$ . As such, volatility decays at half the rate expected by the baseline demographic model. For equation (3) to produce this result, synchrony (pairwise correlations) must decline with size as

(7) $$\rho \left(N\right)\propto N^{-1/2}.$$

Then for large N , the synchrony term dominates:

(8) $$\mathrm{Var}\left(r|N\right)\approx s^{2}\;c_{\rho }N^{-1/2},\Rightarrow \sigma \left(r|N\right)\propto N^{-1/4},$$

matching the results we obtain from the data.

Further, these results suggest populations consist of effective correlated “clusters,” or modular demographic units of average size $m\left(N\right)\propto N^{1/2}$ . The effective number of independent draws is $K\left(N\right)\propto N^{1/2}$ , yielding $\sigma \left(r\right)∼K^{-1/2}∼N^{-1/4}$ . As such, volatility scales negatively with population size at half the rate of the baseline demographic model as pairwise correlations within populations dampen the rate at which stochasticity is averaged out by scale.

### Link to space use and density

Table 3 shows β=3/4 . Since $A\propto N^{3/4}$ , we have $A/N\propto N^{-1/4}$ and so $D=N/A\propto N^{1/4}$ . Substituting into equation (8) we have,

(9) $$\sigma \left(r\right)\propto N^{-1/4}={\left(N/A\right)}^{-1}=D^{-1}.$$

Thus, population stability increases in direct proportion to population density.

### Unified variance model

Conceptually, we can then define a full growth model composed of three terms:

(10) $$r=f\left(\text{individual contributions},\;,\text{demography},\;,\text{synchrony}\right).$$

We then formalize this into a general model that considers the effects of demography and synchrony (pairwise correlations) on population growth:

(11) $$r_{i,t}=\mu_{i}-\phi \log N_{i,t}+\sigma_{d}N_{i,t}^{-1/2}\eta_{i,t}+\sigma_{\rho }N_{i,t}^{-1/4}\xi_{i,t},$$

where ϕ is a density-dependence coefficient and both η and $\xi ∼N\left(0,1\right)$ . The conditional variance is then

(12) $$\mathrm{Var}\left(r|N\right)=\sigma_{d}^{2}N^{-1}+\sigma_{\rho }^{2}N^{-1/2}.$$

The observed scaling of volatility and space use shows that human demography is not reducible to independent births and deaths but is structured by social, ecological and institutional synchrony. Synchrony inflates volatility, but density, sharing and spatial organization counteract synchrony, stabilizing demographic trajectories. Table 4 summarizes this chain of exponents linking space use, correlation and volatility.

**Table 4.** Exponent mapping from spatial overlap to correlation and volatility Table 4. long description.

Quantity	Scaling relation	Implied exponent
Area use (per capita)	$A/N∼N^{-1/4}$	−1/4
Population density	$D=N/A∼N^{1/4}$	1/4
Pairwise correlation (synchrony)	$\rho \left(N\right)∼N^{-1/2}$	−1/2
Variance of growth rate	$\mathrm{Var}\left(r|N\right)∼N^{-1/2}$	−1/2
Volatility (std. dev.)	$\sigma \left(r\right)=N^{-1/4}$	−1/4
Density law of volatility	$\sigma \left(r\right)∼D^{-1}$	−1

### Data limitations and sources of uncertainty

As detailed above, our analyses rely on census time series that are short, irregularly spaced and heterogeneous in quality. Enumeration in remote indigenous regions is subject to logistical constraints, and even small counting errors can disproportionately affect growth rate estimates in small populations. Although random measurement noise may inflate volatility estimates, our inference depends primarily on how volatility scales with population size rather than on absolute variance levels.

It is also important to recognize that populations in the dataset are also not fully spatially independent. Neighboring groups often share climatic regimes, epidemiological exposure and broader ethnolinguistic networks, introducing spatial autocorrelation that may reduce effective sample size and increase parameter uncertainty. While such shared forcing is itself part of the synchrony process we seek to quantify, it complicates strict statistical independence assumptions. Unfortunately, we do not have access to spatial data to control for these effects, which would allow us to examine other aspects of ecological synchrony. However, we can only note that in similar studies of spatial ecology in small-scale human societies, spatial autocorrelation and other potentially confounding factors has had negligible impacts on empirically estimated scaling parameters (Hamilton et al., 2007, 2024).

In addition, demographic shocks, including epidemics, displacement and episodic contact events, introduce nonstationarity into growth rates. These rare but severe events contribute to observed volatility and could disproportionately influence scaling estimates. Finally, the synchrony-extended model assumes exchangeable pairwise correlations within populations, whereas real social systems likely exhibit heterogeneous network structures. The inferred effective modularity should therefore be interpreted as a coarse-grained approximation rather than a literal demographic partition. However, as above, we observe that empirical social network structures in small-scale societies are remarkably consistent in their size, modularity and hierarchical organization (Hamilton et al., 2007, 2020; Buchanan and Hamilton, 2023; Pablo et al., 2022). Despite these limitations, the consistent departure from classical $\sigma \left(r\right)\propto N^{-1/2}$ scaling suggests that independence is systematically violated. While parameter estimates carry uncertainty, the qualitative evidence for demographic synchrony remains robust across populations and regions.

## Discussion

Using census data, we show that demographic synchrony at the individual scale inflates volatility at the population scale. Even numerically large human populations exhibit stochastic demographic fluctuations far greater than predicted under classical assumptions of independence. From an ecological perspective, this result challenges the long-standing premise that increasing population size alone guarantees stability through statistical averaging. Under independent demographic stochasticity, volatility in per-capita growth rates should scale as $\sigma \left(r\right)\propto N^{-1/2}$ . Empirically, however, we observe $\sigma \left(r\right)\propto N^{-1/4}$ . This systematic departure from independence indicates that individuals within populations do not behave as statistically independent demographic units. Instead, social organization induces demographic modularity as individuals are embedded within shared ecological and social environments, coupling their demographic outcomes.

There is no reason to assume that this result is a unique feature of human societies. On the contrary, we suggest it likely reflects a more general property of cooperative systems where cooperation reduces idiosyncratic risk by pooling resources, sharing labor and coordinating reproduction, yet the same mechanisms that buffer individuals against uncertainty introduce correlations among their demographic fates. In statistical terms, cooperation increases covariance while reducing individual variance. Because aggregate population variance depends on both variance and covariance, the latter becomes dominant as cooperation intensifies.

For example, our results suggest that a cooperative population of N=400 individuals behaves demographically as if composed of only $K\left(N\right)\approx 20$ independent demographic units. The effective dimensionality of the population is dramatically reduced. Such dimensional collapse is precisely what one would expect in any cooperative organism in which reproduction, survival or resource acquisition is coordinated within modular social units, whether breeding pairs in wolves (Mech and Boitani, 2003; Smith et al., 2010), cooperative clusters in acorn woodpeckers (Koenig and Mumme, 1987; Joseph and Koenig, 2002; Koenig, 2002), dominant females in meerkat groups (Clutton-Brock et al., 1999, 2001) or colonies of eusocial insects (Bourke and Franks, 1995; Seeley, 2009). In each case, individuals do not experience independent risks; rather, they experience shared exposures.

In the model, pairwise correlation ρ captures the degree to which independence is violated. Ecologically, ρ represents shared environmental forcing and shared social exposure. Traditional, small-scale human populations are spatially clustered, often kin-structured, and socially integrated. Kinship, reciprocity and coordinated subsistence couple births and deaths across households (Hill and Hurtado, 2009; 2017; Gurven et al., 2000; Hill, 2002; Gurven, 2004; Kramer, 2010; Page et al., 2019; Kraft et al., 2021; Kraft et al., 2023). Fission–fusion mobility further synchronizes outcomes, as movement occurs in modular groupings rather than independently (Hill et al., 2011; Hill 2014; Hamilton et al., 2016; 2024). Environmental shocks, such as rainfall variability, prey fluctuations or crop failures, act simultaneously across co-resident households and pathogens propagate through dense interaction networks, synchronizing mortality, as well-documented in waves of post-contact epidemics decimating indigenous populations throughout Amazonia over the past 500 years (Ribeiro, 1967; Bodard, 1974; Hemming, 1978; Hurtado et al., 2001; Vaz, 2011; Hamilton et al., 2014).

These mechanisms of synchrony are general demographic processes in social species. In any cooperative species, shared nesting sites, communal dens, collective provisioning or reproductive skew couple demographic outcomes (Brown, 1987; Koenig and Mumme, 1987; Keller and Reeve, 1994; Creel and Macdonald, 1995; Clutton-Brock et al., 2001; Bourke, 2011). The ecological literature has long recognized that spatial synchrony among populations increases extinction risk by eroding rescue effects. Our findings demonstrate an analogous phenomenon *within* populations, as synchrony among individuals erodes internal stabilizing effects. Just as synchrony among subpopulations elevates regional extinction risk in metapopulations, synchrony among individuals elevates extinction risk within cooperative populations.

The synchrony-extended model formalizes this process. When ρ=0 , volatility declines as $\sigma \left(r\right)∼N^{-1/2}$ ; when ρ>0 , the decay is slower, yielding the empirical scaling $\sigma \left(r\right)∼N^{-1/4}$ . The implied relationship $\rho \left(N\right)∼N^{-1/2}$ suggests that correlations weaken with size, but only gradually. Populations become denser as they grow, with area scaling as $A∼N^{3/4}$ and density $D∼N^{1/4}$ . Increasing density enhances both exposure coupling and buffering capacity. On one hand, shared space increases environmental synchrony; on the other, denser exchange networks redistribute risk. The net empirical outcome is $\sigma \left(r\right)\propto D^{-1}$ , indicating that density weakens synchrony but does not eliminate it. In other words, larger populations are more stable because they are denser (Table 4), but the stabilizing effect of scale is weaker than expected under demographic independence due to increasing synchrony.

This dual role of density reveals the core tension of the cooperation–synchrony paradox. Cooperation evolved as a risk-pooling strategy, reducing individual variance in fitness-related outcomes. Yet cooperation simultaneously synchronizes exposure to large-scale shocks at the population level. Shared subsistence bases, coordinated reproduction and dense interaction networks mean that droughts, epidemics or regional disturbances impact all members simultaneously. Thus, cooperation stabilizes individuals while potentially increasing systemic fragility. This then begs the question: at what ecological scale does cooperation shift from buffering risk to amplifying shared vulnerability? And how does this vary across species populations that vary in their cooperative behaviors?

This trade-off mirrors well-known properties of tightly coupled systems in ecology and complex systems theory: systems that maximize local efficiency through integration often become more sensitive to correlated external shocks (May, 1972; Tilman et al., 1998; Scheffer et al., 2001; Watts, 2002; Loreau and de Mazancourt, 2008; Helbing, 2013). Cooperative populations occupy precisely this structural regime of high internal coupling, shared environmental exposure and reduced effective dimensionality.

**Figure 3.**
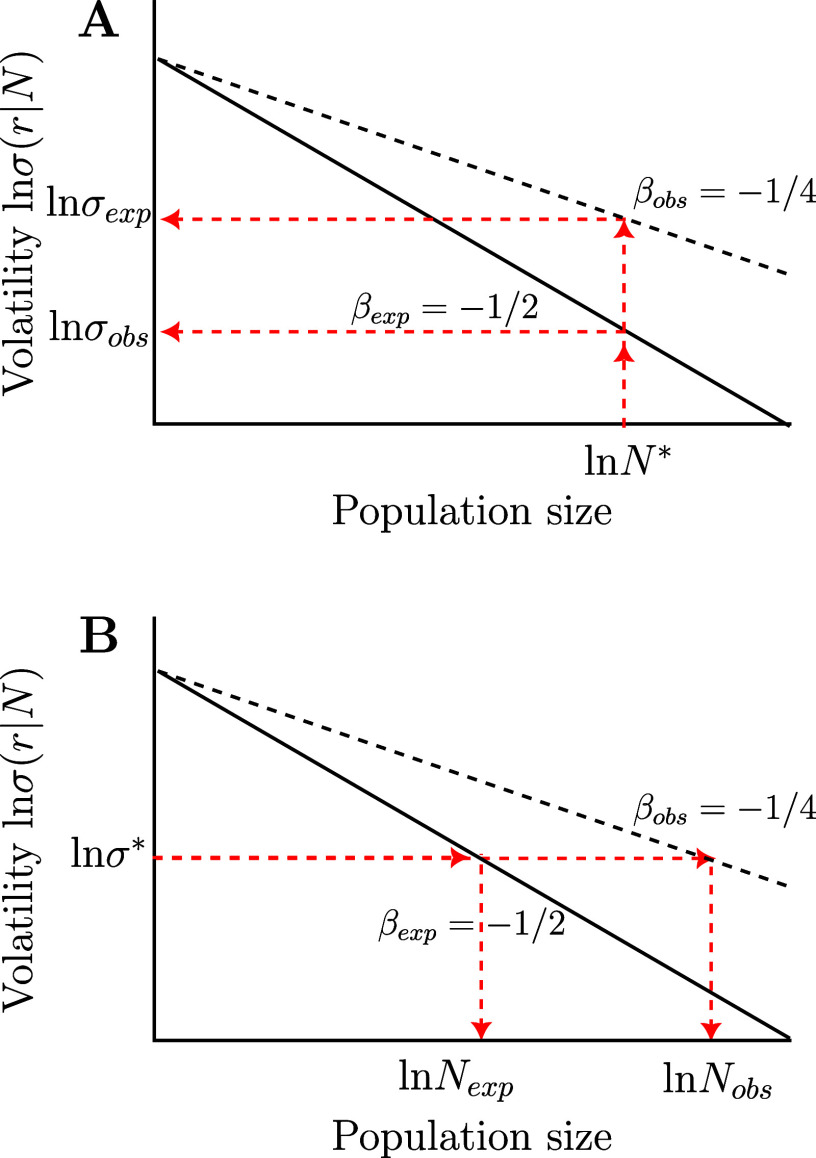
Demographic fluctuations schematic. (A) For a given population size, $N^{*}$ , the expected volatility of the population $\sigma_{\exp}$ is lower than the observed volatility $\sigma_{\mathrm{obs}}$ as the observed slope $\beta_{\mathrm{obs}}=-1/4$ is shallower than the expected slope $\beta_{\mathrm{obs}}=-1/2$ . (B) For a given level of volatility, $\sigma^{*}$ , the expected population size $N_{\exp}$ is lower than the observed population size $N_{\mathrm{obs}}$ . As such, all populations are more volatile than expected for any given size, or equivalently, large populations are observed to be as volatile as smaller populations under the expected baseline demographic model. Figure 3. long description.

The implications for extinction risk are substantial. Classical population viability analyses assume $\sigma^{2}\left(r\right)\propto 1/N$ and thus predict relatively modest minimum viable population sizes (Shaffer, 1981; Dennis et al., 1991; Morris and Doak, 2002; Traill et al., 2007). Under synchrony, however, minimum viable population sizes are roughly the square of baseline estimates. A population predicted to remain viable at $N_{\mathrm{MVP}}=100$ under independence may require 10,000 under synchrony and so populations that appear demographically large enough to be secure may still be ecologically vulnerable (Figure 3B).

## Conclusion

The synchrony-extended model thus formalizes a general ecological mechanism: when demographic events are correlated across individuals due to shared environments and social coupling, the stabilizing effect of size is weakened, volatility declines more slowly with N and extinction risk increases. In this view, the human case we document here is not anomalous but illustrative of a general demographic outcome. It provides a rare quantitative window into a broader ecological law of demography where scale does not guarantee stability when demographic fates are synchronized by shared ecologies and cooperation.

## Supporting information

10.1017/ext.2026.10013.sm001 Hamilton and Walker supplementary material Hamilton and Walker supplementary material

## Data Availability

All data used in this study are available as Supplementary Information attached to this paper.

## Supplementary material

The supplementary material for this article can be found at http://doi.org/10.1017/ext.2026.10013.

## Long descriptions

### Figure 1. Long description

Panel A is a histogram of Frequency versus Population sizes N on a logarithmic x-axis. The blue bars form a bell shape centered around a red vertical line labeled Median N equals 414. An inset histogram shows Mean population size N-bar with a Median N-bar equals 507.

Panel B is a histogram of Frequency versus Growth rate r. The distribution is narrow and peaked around a red vertical line labeled Mean r equals 0.032. An inset histogram shows Mean growth rate r-bar with a Mean r-bar equals 0.039.

Panel C is a scatter plot of Growth rate r versus Population sizes N. Data points are concentrated near zero growth across all population sizes, creating a funnel shape that narrows as N increases. A horizontal red line represents the regression r equals 0.032 plus 0 N, with R-squared much less than 0.01. An inset scatter plot shows Mean growth rate r versus Mean population size N-bar with a similar horizontal trend line.

Panel D is a histogram of Frequency versus Area A in hectares on a logarithmic x-axis. The distribution is approximately lognormal, centered around a red vertical line labeled Median A equals 2095.26.

Navigate back to Figure 1..

### Table 1. Long description

The table presents regression results for the dependent variable r.

* The first row shows the independent variable r-hat sub i comma t with a coefficient of negative 0.0000 and a confidence interval of negative 0.0000 to 0.0000.

* The Constant is 0.03 with three asterisks indicating significance at the p less than 0.01 level, with a confidence interval of 0.03 to 0.04.

* Observations total 1,353.

* R super 2 is 0.0000.

* Residual standard error is 0.12 with 1,351 degrees of freedom.

* F statistic is 0.004 with 1 and 1,351 degrees of freedom.

A note at the bottom defines significance levels as one asterisk for p less than 0.1, two asterisks for p less than 0.05, and three asterisks for p less than 0.01.

Navigate back to Table 1..

### Figure 2. Long description

Panel A is a log-linear scatter plot. The horizontal axis represents Population sizes, N, ranging from 1 to 100,000 on a logarithmic scale. The vertical axis represents Volatility, sigma-hat open parenthesis r given N sub bin close parenthesis, ranging from 0.01 to 0.30. Blue data points show a downward trend from top-left to bottom-right. A solid red regression line passes through the points with a gray shaded confidence interval. The equation sigma-hat open parenthesis r given N sub bin close parenthesis equals 0.46 N sub bin to the negative 0.26 power, with an R-squared of 0.93, is displayed in the plot area.

Panel B is a log-log scatter plot. The horizontal axis represents Population size, N, from 1 to 100,000. The vertical axis represents Area, A, in kilometers-squared, from 1 to 100,000. Blue data points are clustered and show an upward trend. A solid red regression line with a gray shaded confidence interval indicates a sublinear increase. The equation A equals 0.20 N to the 0.75 power, with an R-squared of 0.39, is displayed in the top-left of the plot area.

Navigate back to Figure 2..

### Table 2. Long description

The table presents regression results for the dependent variable sigma-hat open parenthesis r vertical bar N sub bin close parenthesis.

Row 1: alpha has a coefficient of negative 0.26 with three asterisks, and a confidence interval of negative 0.30 to negative 0.21.

Row 2: natural log of c has a coefficient of negative 0.81 with three asterisks, and a confidence interval of negative 1.09 to negative 0.53.

Row 3: Observations total 13.

Row 4: R-squared is 0.93.

Row 5: Residual standard error is 0.20 with 11 degrees of freedom.

Row 6: F statistic is 142.78 with three asterisks and degrees of freedom 1 and 11.

A footer note indicates significance levels: one asterisk for p less than 0.1, two asterisks for p less than 0.05, and three asterisks for p less than 0.01.

Navigate back to Table 2..

### Table 3. Long description

The table consists of two columns. The first column lists the variables and the second column provides the values for the dependent variable l n A.

* Row 1: The dependent variable is l n A.

* Row 2: beta has a value of 0.75 with three asterisks, followed by a range of 0.61 to 0.90 in brackets.

* Row 3: Constant has a value of 2.99 with three asterisks, followed by a range of 2.07 to 3.90 in brackets.

* Row 4: Observations is 160.

* Row 5: R-squared is 0.39.

* Row 6: Residual standard error is 1.45 with degrees of freedom equal to 158.

* Row 7: F statistic is 100.94 with three asterisks and degrees of freedom equal to 1 and 158.

A footnote indicates that one asterisk means p is less than 0.1, two asterisks mean p is less than 0.05, and three asterisks mean p is less than 0.01.

Navigate back to Table 3..

### Table 4. Long description

The table consists of three columns: Quantity, Scaling relation, and Implied exponent.

* Row 1: Area use per capita. Scaling relation is A forward slash N is proportional to N to the negative 1 forward slash 4 power. Implied exponent is negative 1 forward slash 4.

* Row 2: Population density. Scaling relation is D equals N forward slash A which is proportional to N to the 1 forward slash 4 power. Implied exponent is 1 forward slash 4.

* Row 3: Pairwise correlation synchrony. Scaling relation is rho open parenthesis N close parenthesis is proportional to N to the negative 1 forward slash 2 power. Implied exponent is negative 1 forward slash 2.

* Row 4: Variance of growth rate. Scaling relation is V a r open parenthesis r vertical bar N close parenthesis is proportional to N to the negative 1 forward slash 2 power. Implied exponent is negative 1 forward slash 2.

* Row 5: Volatility standard deviation. Scaling relation is sigma open parenthesis r close parenthesis equals N to the negative 1 forward slash 4 power. Implied exponent is negative 1 forward slash 4.

* Row 6: Density law of volatility. Scaling relation is sigma open parenthesis r close parenthesis is proportional to D to the negative 1 power. Implied exponent is negative 1.

Navigate back to Table 4..

### Figure 3. Long description

The figure consists of two panels, A and B, both plotting Volatility l n sigma open parenthesis r vertical bar N close parenthesis on the y-axis against Population size on the x-axis. Both panels feature a solid line representing the expected slope beta sub e x p equals negative 1 over 2 and a dashed line representing the observed slope beta sub o b s equals negative 1 over 4.

Panel A shows a fixed population size l n N super asterisk on the x-axis. A vertical red dashed arrow extends upward from this point, intersecting the solid line first and then the dashed line. Horizontal red dashed arrows point from these intersections back to the y-axis, showing that for a given size, the observed volatility l n sigma sub o b s is higher than the expected volatility l n sigma sub e x p.

Panel B shows a fixed volatility level l n sigma super asterisk on the y-axis. A horizontal red dashed arrow extends to the right, intersecting the solid line first and then the dashed line. Vertical red dashed arrows point down from these intersections to the x-axis, showing that for a given volatility, the observed population size l n N sub o b s is larger than the expected population size l n N sub e x p.

Navigate back to Figure 3..
