# Supplementary material for: Demographic synchrony increases volatility and extinction risk in human populations
Source: Camb Prism Extinct. 2026 May 5;4:e8. doi: 10.1017/ext.2026.10013 (PMC13430546; doi:10.1017/ext.2026.10013)
Supplement: Peer review history

# Reviewer report: Review: Demographic synchrony increases volatility and extinction risk in human populations — R0/PR2

**DOI:** 10.1017/ext.2026.10013.pr2

**Authors:** July Pilowsky

**Affiliations:** Cary Institute of Ecosystem Studies, United States

This is a paper that uses empirical data to show that demographic synchrony in traditional human societies increases the volatility of population growth more than would be expected for population size by effectively reducing the number of demographic units, thereby increasing the effect of demographic stochasticity. I found this paper very interesting. I bring my perspective as an ecologist who has researched cooperatively breeding non-human animals, which I hope will be of some help to you as anthropologists.

What I found exciting about this paper as an ecologist was your proposed cooperation-synchrony paradox, which proposes a trade-off between the benefits of cooperation and the costs of demographic synchrony. The key point in my view, which you mention in lines 51-53 on page 14, is that cooperation decreases temporal volatility of demographic rates for individuals, but increases the temporal volatility of population growth for the whole population. This population-level volatility has important implications for extinction risk. What I propose to you is that all of the points you’re making could be informed by, and could inform, research on cooperatively-breeding non-human animals. Humans are just another cooperatively breeding mammal, after all, and I think this paper would both benefit from comparison to relevant studies in other cooperatively breeding animals, and would contribute to the body of research on extinction risk in cooperatively breeding vs. non-cooperative non-human animals. I’m already excited to find out whether the power laws you’ve found about volatility hold true in other cooperatively breeding organisms.

Since you’re anthropologists, I’m happy to point you toward some research in ecology that would enrich your discussion of your own results, since I’m very familiar with this literature.

Rubenstein, D. R., & Lovette, I. J. (2007). Temporal environmental variability drives the evolution of cooperative breeding in birds. Current Biology, 17(16), 1414-1419.

Rubenstein, D. R. (2011). Spatiotemporal environmental variation, risk aversion, and the evolution of cooperative breeding as a bet-hedging strategy. Proceedings of the National Academy of Sciences, 108(supplement_2), 10816-10822.

Koenig, W. D., & Walters, E. L. (2015). Temporal variability and cooperative breeding: testing the bet-hedging hypothesis in the acorn woodpecker. Proceedings of the Royal Society B: Biological Sciences, 282(1816), 20151742.

I think contextualizing your results within this literature would improve your discussion and also attract the attention of ecologists interested in extinction risk and population volatility in non-human animals.

Something in your paper I wanted to interrogate was your discussion of individual space use shrinking as density increases. You all are the anthropologists, not me, but my understanding of hunter-gatherer societies is that individual space use is not as common as space use on the level of the family, clan, or band. So is the shrinking and overlap of individual space use the key point here, or the shrinking and overlap of family/band/clan space use?

I have some questions about the methods section here. First, I have some amount of concern about you lumping in population growth rates into the same bin of population size without any thought to spatial structure and spatial autocorrelation. You haven’t provided a map or even a sense of the geographical area covered by your data (you should, by the way), but Brazil is a large country and I would expect populations near each other to have more similar growth rates than populations far away from each other. Can you test for spatial autocorrelation in your growth rates? Account for it in your analysis? Seems like it could be important.

My other point, as I just alluded to, is that I want more information about your data sources. What is the geographic extent? Is the distribution even, or are there areas with more data points than others? How was the population estimated? Was it by government administrators, or were population size estimates given by the societies themselves?

I had some concerns about the structure of this paper. First of all, you state results at the end of the introduction, which is not the convention. Also, you give information about your dataset in the results, when typically you report on the dataset you analyzed in the methods.

With these broader comments said, here are some more fine-grained comments:

1) Page 3, Line 11: I was surprised to see you citing the popular science book Collapse, especially since the book created such an uproar among anthropologists that a veritable fleet of articles, essays, and books refuting its claims arose in response to it.

2) Page 7, Line 44: How is territory size estimated? You never reviewed this in the methods. It’s not a trivial problem, because traditional societies, unlike modern nation-states, do not have strict borders.

3) Page 7, Line 45: You refer to an inset in Figure 1D, but there is no inset in that panel.

4) Page 14, Lines 29-36: I’m confused about what your argument is exactly. Are you saying that cooperation increases volatility when density is low but decreases it when density is high? Why?

# Editor report: Recommendation: Demographic synchrony increases volatility and extinction risk in human populations — R0/PR3

**DOI:** 10.1017/ext.2026.10013.pr3

**Affiliations:** The University of Adelaide, Australia

I apologise for the time that this paper has been in review, but securing reviewers has been challenging. The paper has now been examined by an external reviewer and myself. We both think that the paper is interesting and important, but needs to address some key issues regarding structure and detail.

Like Reviewer 1 I found the structure of the paper to be unusual. I would like to see the Results section rewritten so that methods are in the Methods section and results in the Results section. Please refer to other papers in Extinctions. The Conclusion could also be much more concise. 

I would like to see a greater emphasis on how the spatial and temporal structure of the data could influence the results. Currently, there is no discussion of the impact of data uncertainty. Accordingly, it would be good to do some testing of model fit using cross validation or something similar. This would help increase confidence in the findings.

Lastly, to increase the broad appeal of the article I would also like to see the ecological literature better integrated into the Introduction and Discussion of the paper. This is because there are lots of parallels. 

# Editor report: Decision: Demographic synchrony increases volatility and extinction risk in human populations — R0/PR4

**DOI:** 10.1017/ext.2026.10013.pr4

**Affiliations:** Biological Sciences, The University of Queensland Faculty of Science, Australia

No accompanying comment.

# Reviewer report: Review: Demographic synchrony increases volatility and extinction risk in human populations — R1/PR6

**DOI:** 10.1017/ext.2026.10013.pr6

**Authors:** July Pilowsky

**Affiliations:** Cary Institute of Ecosystem Studies, United States

This revision is a major improvement over the original manuscript. It foregrounds the trade-off in how cooperative behavior increases individual stability while increasing population volatility, and explores the possible implications of this trade-off for the variance in cooperative behavior across populations (Page 18 Line 38). Here are the areas where I asked for improvement in the last version and how the authors delivered:

1) Better contextualization of their results with respect to non-human animals. The authors did an excellent job here, comparing bet-hedging and stability in humans with that in cooperative meerkats, wolves, and acorn woodpeckers.

2) More detail on the dataset and its limitations. It is now clear how the data were obtained, what the data can tell us, and what they cannot. I can appreciate both the strengths and weaknesses of these data relative to other datasets on human demography.

3) The effect of population density on population volatility and how it interacts with demographic synchrony is now much better explained.

I hope that this research will spark comparative investigations of demographic synchrony in other cooperatively breeding populations, both human and non-human, as well as considerations of this issue in population viability analyses of vulnerable cooperative breeders.

# Editor report: Recommendation: Demographic synchrony increases volatility and extinction risk in human populations — R1/PR7

**DOI:** 10.1017/ext.2026.10013.pr7

**Affiliations:** The University of Adelaide, Australia

The manuscript was returned to one of the original reviewers. They concluded that all of their main suggestions had been addressed, recommending acceptance of the paper. I agree that the paper is now ready for publication.

# Editor report: Decision: Demographic synchrony increases volatility and extinction risk in human populations — R1/PR8

**DOI:** 10.1017/ext.2026.10013.pr8

**Affiliations:** Biological Sciences, The University of Queensland Faculty of Science, Australia

No accompanying comment.
